# Case Report: Herpes Simplex Virus Type 2 Acute Retinal Necrosis With Viral Encephalitis in Children

**DOI:** 10.3389/fmed.2022.815546

**Published:** 2022-03-16

**Authors:** Luyao He, Jialiang Duan, Qingli Shang

**Affiliations:** Department of Ophthalmology, The Second Hospital of Hebei Medical University, Shijiazhuang, China

**Keywords:** herpes simplex virus-2, immunocompetent population, viral encephalitis, vitreous opacity, retinal necrosis

## Abstract

**Background:**

Few cases concerning acute retinal necrosis with viral encephalitis in children have been reported, especially cases where the fundus cannot be identified due to severe vitreous opacity in the early stage that makes diagnosis difficult.

**Methods:**

We conducted a retrospective review of an unusual case of herpes simplex virus-2 (HSV-2) acute retinal necrosis with viral encephalitis in an immunocompetent child, along with a review of relevant literature published up to September 2021.

**Result:**

An 11-year-old girl presented with an approximate 20-day history of ocular redness and decreased visual acuity in the left eye. Examination revealed anterior uveitis and vitreous opacity in the left eye. An anterior chamber tap was performed because the fundus could not be observed clearly, and the aqueous humor was positive for HSV-2 DNA. Cerebrospinal fluid also tested positive for HSV-2. She was diagnosed with acute retinal necrosis syndrome and viral encephalitis. The condition was controlled with timely antiviral and steroid therapy. She was also treated with prophylactic laser therapy to prevent retinal detachment during subsequent follow-up. The pathogenesis, diagnosis, and treatment of HSV-2 acute retinal necrosis in children and the association between acute retinal necrosis and viral encephalitis are further discussed, based on published literature.

**Conclusion:**

HSV-2-related pediatric acute retinal necrosis may be due to the acquisition of subclinical infection with HSV-2 during parturition, followed by reactivation of the virus latent in the body on account of certain factors. Moreover, it may be complicated with viral encephalitis. For suspected cases with invisible fundus, early intraocular fluid examination is especially helpful for differential diagnosis. Early diagnosis, early treatment, and timely prophylactic laser treatment to prevent retinal detachment are key to a better prognosis. Physicians need to pay attention to such suspected cases during diagnosis and treatment.

## Introduction

Acute retinal necrosis (ARN) syndrome is a viral infectious disease, clinically characterized by severe and extensive uveitis, vitritis, retinal vasculitis, retinal necrosis, and retinal detachment, which in combination indicate an advanced disease stage (1). ARN has an acute onset and rapid progression, and can develop at all ages. However, ARN is relatively more common in adults than in children (2). Here, we present a child with ARN, viral encephalitis, and herpes simplex virus-2 (HSV-2) infection and a literature review of HSV-2 ARN in children.

## Materials and Methods

We present a case report of ARN with viral encephalitis in an immunocompetent child with HSV-2 infection. We also performed a PubMed database search of relevant publications in English up to September 2021, using the following keywords: “acute retinal necrosis,” “children,” and “herpes simplex virus” joined by the operator “AND.” We evaluated and reviewed all relevant literature and relevant reported cases.

## Case Description

**Figure 1:**
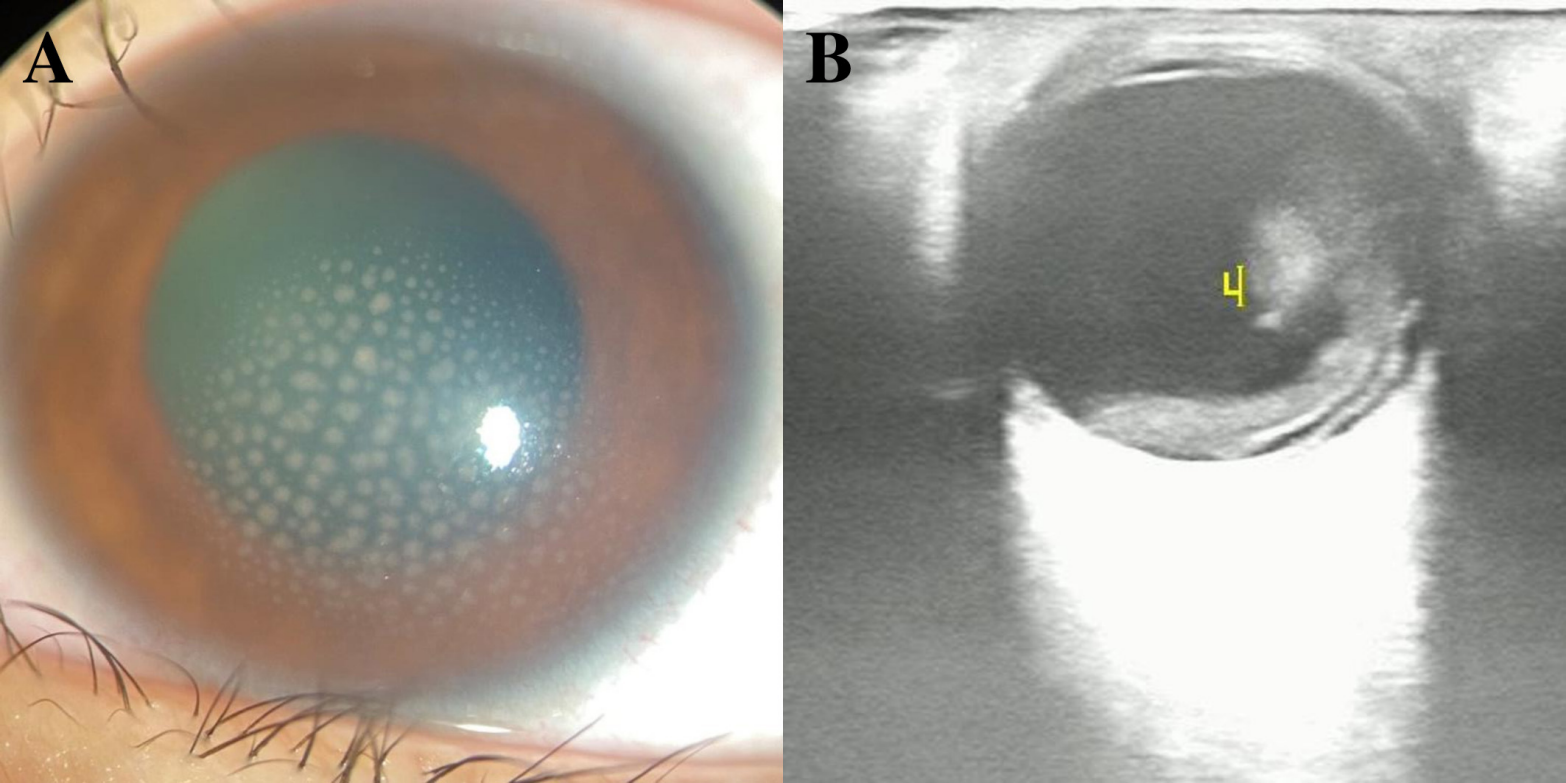
**(A)** Slit-lamp examination revealed mutton-fat keratic precipitates in the middle and below the corneal endothelium and 3+ cells and 3 + flare in the anterior chamber. **(B)** Ultrasonography revealed severe vitreous opacity, posterior vitreous detachment in the left eye, and thickening of the posterior wall of the left eyeball.

**Figure 2:**
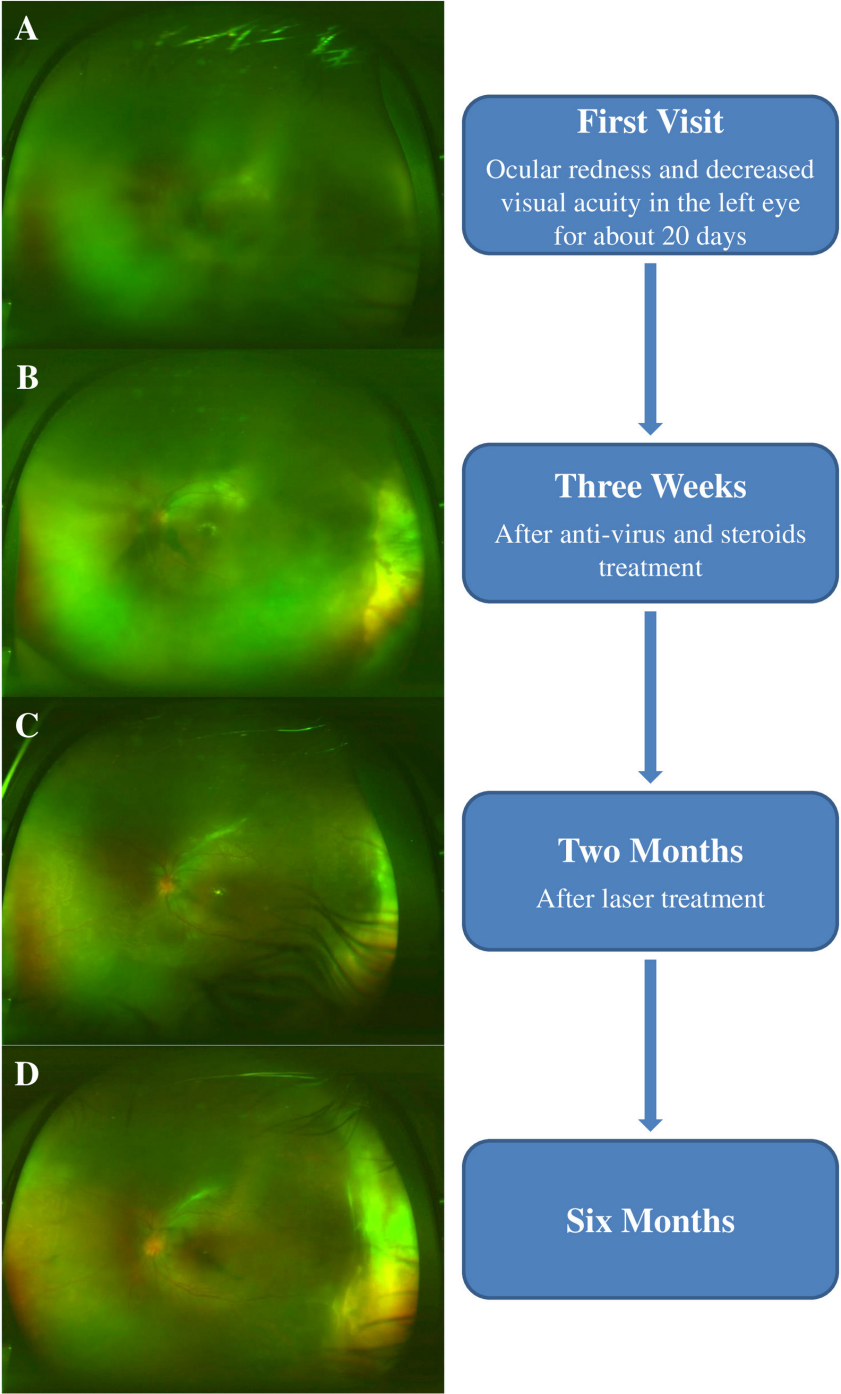
**(A)** At the initial examination, the fundus could not be inspected due to vitreous opacity. **(B)** After 3 weeks of antiviral and steroid treatment, the fundus could be gradually observed. Wide-field fundus photography revealed optic disc edema, macular edema, and peripheral retinal necrosis. **(C)** At the 2-month follow-up, peripheral retinal proliferation was observed in the fundus examination; thus, the patient was provided prophylactic laser treatment. **(D)** At the last follow-up, wide-field fundus photography revealed the attachment of the retina.

An 11-year-old girl was admitted to our hospital with an approximate 20-day history of ocular redness and decreased visual acuity in the left eye. On admission, her best-corrected visual acuity (BCVA) was 20/20 in the right eye, and hand motion in the left eye. The patient's intraocular pressures were 19 mmHg and 28 mmHg in the right and left eyes, respectively. Slit-lamp examination revealed conjunctival hyperemia, mild corneal edema, mutton-fat keratic precipitates in the middle and below the corneal endothelium, 3+ cells and 3+ flare in the anterior chamber (Figure 1A), and severe vitreous opacity in the left eye. The fundus could not be inspected because of the vitreous opacity (Figure 2A). Slit-lamp and fundus examinations of her right eye were unremarkable. Ultrasonography showed severe vitreous opacity, posterior vitreous detachment in the left eye, and thickening of the posterior wall of the left eyeball (Figure 1B). Serological test results for toxoplasmosis, rubella, cytomegalovirus (CMV), and herpes simplex virus indicated that the patient tested positive for HSV-2 immunoglobulin G (IgG), CMV IgG, and rubella virus IgG, and tested negative for other infections. The results of routine blood tests, liver function, renal function, syphilis, tuberculosis, and other laboratory tests showed no significant abnormalities. Granulomatous panuveitis was considered, and differential diagnoses included viral or bacterial infection and toxoplasmosis or *Toxocara canis* infection. After informed consent was obtained from her parents, a left eye anterior chamber tap was performed. The aqueous humor results were positive for HSV-2 DNA. Brain magnetic resonance imaging (MRI) scans showed abnormal signal intensity in the right temporal lobe, and dilatation of the right temporal horn when compared with the left temporal horn. After consultation with pediatricians, we performed a lumbar puncture and the cerebrospinal fluid tested positive for HSV antibodies.

According to Classification Criteria for Acute Retinal Necrosis Syndrome (3) key diagnostic criteria, our patient was diagnosed with ARN. She was also diagnosed with viral encephalitis (HSV-2), based on positive test results for HSV antibodies in the cerebrospinal fluid and on brain MRI results. Treatment included administration of intravenous acyclovir and dexamethasone, local steroid eye drops, and mydriasis eye drops. In addition, she received intravitreal ganciclovir injections twice a week for 2 weeks.

**Figure 3:**
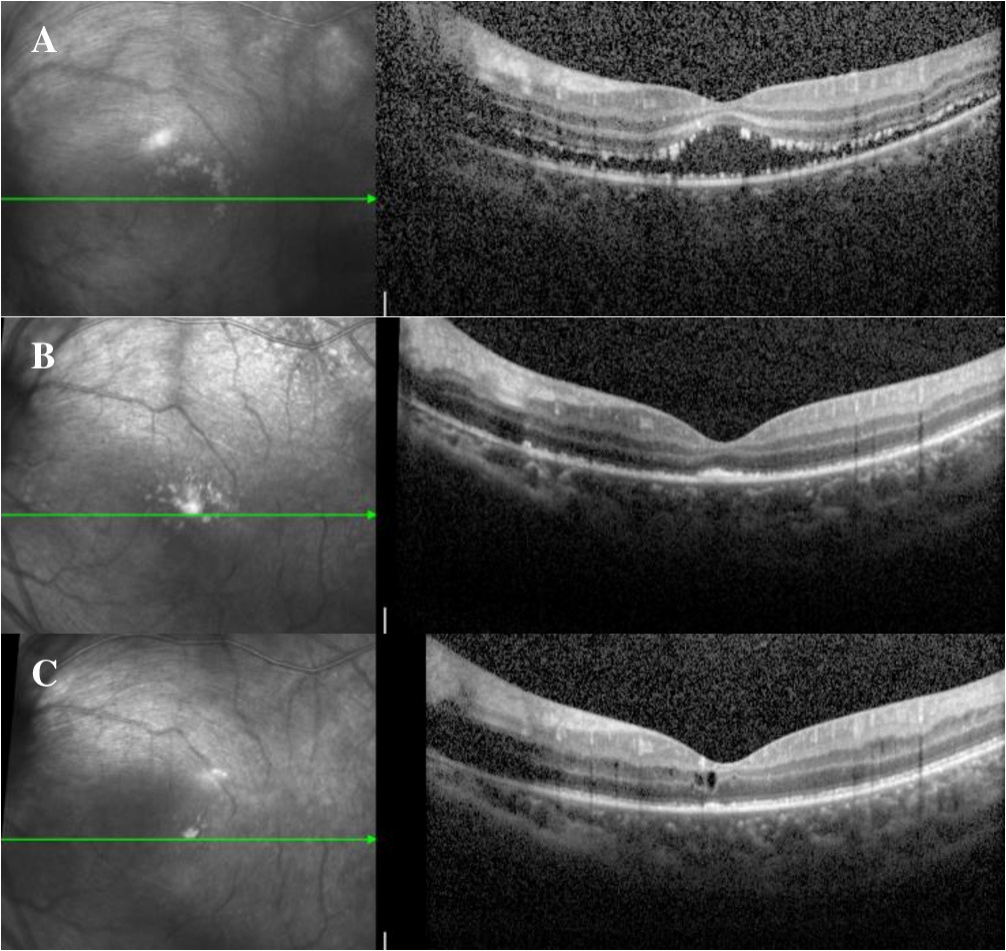
**(A)** After 3 weeks of antiviral and steroid treatment, the fundus could be gradually observed. OCT revealed macular edema. **(B)** At the 2-month follow-up, OCT revealed the attached retina after the patient underwent prophylactic laser treatment. **(C)** At the last follow-up, OCT revealed the retina to be attached; however, cystoid macular edema was still present. OCT, Optical coherence tomography.

Following 3 weeks of treatment, the anterior inflammation was under control, and the left fundus could be gradually observed. Optical coherence tomography (OCT) and wide-field fundus photography showed optic disc edema, macular edema, and peripheral retinal necrosis (Figures 2B, 3A). The recommended time for antiviral therapy has been reported to be between 12 and 14 weeks (4). However, long-term use of antiviral drugs in children may have side-effects such as nephrotoxicity, gastrointestinal upset, and skin lesions (5). Further to a conversation with the patient's parents, they consented to her continuing prophylactic anti-viral therapy; and renal function, blood routine results were closely monitored. She was discharged with a prescription for oral steroids and valacyclovir.

At 2 months, peripheral retinal proliferation was observed in the fundus examination, and the patient was administered prophylactic laser treatment (Figures 2C, 3B). Steroids and valacyclovir were discontinued after 3 months. At the last follow-up, her BCVA was 20/100 in the left eye, and the retina was attached but still had cystoid macular edema on OCT (Figures 2D, 3C).

## Discussion

ARN syndrome is a serious viral infectious disease, and its main pathogenic viruses are varicella zoster virus (VZV), herpes simplex virus types 1 or 2, CMV, and the Epstein-Barr virus (EBV) (6–8). ARN can occur in both adults and children; however, it occurs more commonly in adults, with an average age of onset reported to be between 20 and 60 years (9). Several studies have reported that adult ARN is mainly due to VZV and HSV-1 (10). In contrast, there are significantly fewer reports related to pediatric ARN, and the main causative virus has been reported to be HSV-2 (11–13).

We reviewed the relevant literature concerning ARN, and identified 14 cases of pediatric ARN due to HSV-2 (10, 14–23). Pediatric ARN due to HSV-2 was first reported in 2001 (14). Details concerning 15 cases (including the present case) are summarized in Table 1. The median age of the 15 children was 6.59 years (range, 23 days to 14 years) with no sex predominance (7 boys; 8 girls). Of these 15 patients, 11 patients had unilateral disease and 4 patients had bilateral disease. The fundus of one patient could not be observed due to synechiae between the pupil and the lens, whereas our patient had severe vitreous opacity resulting in an inability to observe the fundus. All the other patients' fundi could be observed, aiding diagnosis. Five patients had combined viral encephalitis (33.3%), and one patient had been diagnosed with neonatal HSV meningoencephalitis after birth and had developed ARN when she was 9 years old. Seven patients had retinal detachment complications (46.7%). Eleven patients were immunocompetent, except for one patient whose information was not available; thus, her immune status could not be determined. Moreover, three patients were immunocompromised due to prematurity. Seven patients had a clear history of neonatal HSV-2 infection, Six patients' mothers had a history of HSV-2 infection, and 10 patients had other herpes virus infection-like manifestations prior to ARN.

**Table 1:** Published cases of pediatric ARN with HSV-2.

**References**	**Age**	**Sex**	**Affected eye**	**Initial visual acuity**	**Whether the fundus could be seen in the early stage**	**Immune status**	**Neonatal herpes infection**	**Other systemic illnesses**	**Whether it was complicated with meningitis or encephalitis**	**Treatment**	**Compli-** **cations**	**Prognosis**
Tan et al. (14)	9 yr	F	Right	20/60	YES	Immunocompetent	N/A	Annual recurrences of right earlobe blistering since 5	NO	Systemic antiviral	NO	20/40
Tan et al. (14)	10 yr	M	Left	20/30	YES	Immunocompetent	N/A	Cold sores	HSV encephalitis	Systemic antiviral	RD	CF
Tran et al. (15)	11 yr	M	Left	CF (OD)	YES	Immunocompetent	N/A	Oral herpes at 3 months, RD (OD) at 9, extracapsular cataract extraction	NO	Systemic and topical antiviral and steroid	NO	20/40
				HM (OS)								
Landry et al. (16)	9 yr	F	Left	HM	NO (Posterior synechiae)	Immunocompetent	YES^*^	Neonatal HSV meningoencephalitis	NO	Systemic antiviral, topical steroid, vitrectomy with scleral buckle, laser	NO	20/60
Khurana et al. (17)	14 yr	F	Right	CF (OD)	YES	Immunocompetent	N/A	Perinatal herpetic keratitis (OS)	NO	Systemic antiviral and steroid, laser	RD	20/50
				20/200 (OS)								
Khurana et al. (17)	10 yr	F	Left	20/40	YES	Immunocompetent	Yes	HSV-positive vesicular scalp dermatitis at 8-day-old	NO	Systemic and topical antiviral	RD	20/200
Charles (10)	8 yr	F	Right	N/A	YES	N/A	Maybe	Developmental delay	NO	Systemic antiviral	NO	N/A
King et al. (18)	9 yr	F	Left	20/200	YES	Immunocompetent	Maybe^*^	NO	NO	Systemic and topical antiviral, systemic steroid	Vitreous hemorrhage, RD	20/40, RAPD, a central posterior subcapsular cataract
Chiquet et al. (19)	4 yr	M	Right	20/200	YES	Immunocompetent	Maybe	Axial muscular hypotony at 2 months, chickenpox at 3, a right non-granulomatous panuveitis at 5 months	NO	Systemic antiviral and steroid, topical steroid and cycloplegic	RD	N/A
Tanaka-Kitajima et al. (20)	3 yr	M	Left	20/50	YES	Immunocompetent	Maybe^*^	Periocular trauma	NO	Systemic antiviral and steroid	NO	20/20
Gupta et al. (21)	25 days	M	Both	N/A	YES	Immunocompromised	YES^*^	Premature, twin birth, twin brother died, crusted skin eruptions with ulceration	HSV encephalitis	Systemic antiviral, laser	RD(OS)	N/A
Gupta et al. (21)	25 days	M	Both	N/A	YES	Immunocompromised	YES^*^	Premature, twin birth	Encephalitis	Systemic antiviral	dead	dead
Hsu et al. (22)	29 weeks	F	Both	N/A	YES	Immunocompromised	YES^*^	Premature, dichorionic diamniotic twins, a vesiculopapular rash at birth	NO	Systemic antiviral, laser	RD	Macular pigmentary disturbances, mild optic atrophy
Ren et al. (23)	23 days	M	Both	N/A	YES	Immunocompetent	YES	Bulging fontanel, nuchal rigidity, skin lesions	HSV-2 encephalitis	Systemic antiviral, intravitreal ranibizumab injection	NO	N/A
Present case (2021)	11 yr	F	Left	HM	No (Severe vitreous opacity)	Immunocompetent	NO	NO	HSV encephalitis	Systemic and topical antiviral and steroid, cycloplegic, laser	NO	20/150

*N/A, Not available*.

* *Mother had a determined history of HSV-2 infection. RD, retinal detachment; RAPD, relative afferent pupillary defect; HSV, herpes simplex virus*.

HSV-2 ARN is similar to ARN, due to viruses such as HSV-1 and VZV, in terms of clinical manifestations, treatment, and prognosis in children. However, these two conditions also differ slightly. HSV ARN is mostly associated with viral encephalitis compared with VZV ARN (11, 24) (Table 1). However, after a review of the case summaries, our findings indicated that 11 of 15 patients with HSV-2 ARN were immunocompetent (Table 1), which corroborated the reported claim that patients with HSV ARN are usually immunocompetent (6). However, some studies have shown that patients with VZV, CMV, and EBV ARN may be in an immunosuppressed state in association with diseases such as human immunodeficiency virus infection, congenital heart disease, and leukemia (25, 26).

ARN can be due to direct viral infection or reactivation of a latent virus, or can occur secondary to diseases such as viral encephalitis, varicella, and herpetic keratitis (27). HSV-2 is mainly transmitted sexually. Direct infection in childhood is relatively rare, and neonatal infection often results from vertical maternal transmission. In this case, the child had no history of HSV-2 infection. For children without a known history of neonatal HSV-2, the pathogenesis of HSV-2 ARN has not been clarified. The generally accepted mechanism is that newborns are infected with HSV-2 at delivery through the birth canal in parturients with genital herpes. The virus may enter the body through sensory nerves located in the conjunctiva or nasal epithelium and travel in neural tissues, such as the olfactory and trigeminal nerves (10). The virus lies latent in newborns but does not cause symptoms of infection, and newborns remain in a state of subclinical HSV-2 infection. At some future point, latent infection can escalate to overt infection due to certain triggers, such as periocular trauma (20), neurosurgery, high-dose corticosteroids, and immunosuppressive agents (24). The virus reactivates and travels to the eye, causing ARN.

Viral encephalitis can be caused due to the reactivation of HSV-2 in the brain or through a retrograde passage of the virus activated in the eye to the brain (28). ARN has been found to be associated with viral encephalitis in several studies. According to previous literature, approximately 13.5% of patients with ARN have combined viral encephalitis, of which 57.1% involves herpes simplex encephalitis (29). Moreover, this proportion also appears to be high among children. Of 15 pediatric patients with HSV-2 ARN, five (33.3%) were found to have viral encephalitis after a retrospective analysis of the relevant cases (14, 21, 23) (Table 1). This may be related to the fact that children have lower immunity than adults, and that they cannot accurately describe the symptoms in time, thus increasing the incidence of complications. Therefore, ophthalmologists should be aware of whether patients have central nervous system infections especially when diagnosing and treating children with ARN.

ARN syndrome generally has a poor prognosis (9) and can quickly affect the other eye if left untreated (30). Early diagnosis and therapy are key to maintaining vision and preventing the involvement of the other eye (16). The diagnostic criteria for ARN include single or multiple retinal necrosis lesions (22). However, in this case, it had not been previously determined that our patient had severe vitreous opacity in the early stage as her fundus could not be observed, which posed a major challenge to our diagnosis. After a timely anterior chamber tap, the diagnosis was confirmed. We consider that the cause of severe vitreous opacification in this patient was possibly due to her normo-immune system. After the virus is activated in the eye, the immune system begins to function. Immune cells engulf the virus (31) and activate other associated cells (32). They also produce cytokines and mediate inflammatory reactions (33), which cause severe intraocular inflammatory reactions and severe vitreous opacification. Therefore, for suspected cases with invisible fundus, early intraocular fluid examination is especially helpful for the differential diagnosis.

The basic treatment methods for ARN mainly include systemic antiviral, anti-inflammatory, and antithrombotic therapy, and local application of cycloplegic eye drops (8). Currently, there are no clear criteria for the treatment of ARN in children although treatment in children has been similar to that in adults. In this case, the child was mainly administered a systemic intravenous infusion and intravitreal injection of drugs in the left eye as part of antiviral therapy, and steroids as part of anti-inflammatory therapy. Early and comprehensive treatment promptly inhibited further retinal damage, and prevented involvement of the other eye.

ARN complications have been found to mainly include retinal detachment, with a general detachment rate in patients with ARN of 49% (34). Retinal detachment was also observed in seven (46.7%) of the 15 pediatric cases of ARN due to HSV-2 in our study (14, 17–19, 21, 22), as shown in Table 1. Once retinal detachment occurs in children, it predisposes them to more serious consequences than those occurring in adults. There can be several reasons why children may present late to an eye clinic after a long period of retinal detachment. In such cases, at presentation, the lesions in these children are prone to involve the macula, accompanied with serious complications, such as proliferative vitreoretinopathy. Prophylactic laser treatment at the earliest possible time has been reported to significantly reduce the risk of retinal detachment in ARN (35, 36). However, it remains to be elucidated whether prophylactic laser treatment is fully effective in preventing retinal detachment (2). In our young patient, prophylactic laser treatment was performed in time to prevent retinal detachment when the child was followed up for 2 months, which enabled subsequent stabilization and recovery. However, the long-term effects of prophylactic laser treatment in preventing retinal detachment in this young patient require further follow-up.

In conclusion, we report a case of ARN with viral encephalitis in an immunocompetent child with HSV-2 infection. The pathogenesis of HSV-2 ARN in children may be due to the acquisition of subclinical infection with HSV-2 during parturition, followed by reactivation of the latent virus in the body due to certain factors. The unique feature of this case was that our patient had severe vitreous opacity in the early stage, making the fundus unobservable, which made diagnosis challenging. Early diagnosis was confirmed using a timely anterior chamber tap. Early administration of antiviral drugs for comprehensive treatment curbed the development of the disease, prevented involvement of her other eye, and achieved a good therapeutic effect. A subsequent prophylactic laser retinopexy may have helped prevent a retinal detachment in the affected eye. This outcome suggests that early diagnosis, early treatment, and subsequent prophylactic laser treatment may improve the prognosis of pediatric ARN. Moreover, in the process of diagnosis and treatment, it is particularly important to assess the presence of central nervous system infection. The low incidence of pediatric ARN increases the chances of this disease being missed during diagnosis and treatment by both pediatricians and ophthalmologists.

## Data Availability Statement

The original contributions presented in the study are included in the article, further inquiries can be directed to the corresponding authors.

## Ethics Statement

The studies involving human participants were reviewed and approved by the Second Hospital of Hebei Medical University. Written informed consent to participate in this study was provided by the participant's parents. Written informed consent was obtained from the participant's parents for the publication of any potentially identifiable images or data included in this article.

## Author Contributions

LH consulted literature, curated the data, and drafted the manuscript. QS diagnosed, treated, and followed up our patient. JD diagnosed and treated our patient, proposed the idea for the case report, followed up our patient, collected photos, and reviewed the article. All authors contributed to the article and approved the submitted version.

## Conflict of Interest

The authors declare that the research was conducted in the absence of any commercial or financial relationships that could be construed as a potential conflict of interest.

## Publisher's Note

All claims expressed in this article are solely those of the authors and do not necessarily represent those of their affiliated organizations, or those of the publisher, the editors and the reviewers. Any product that may be evaluated in this article, or claim that may be made by its manufacturer, is not guaranteed or endorsed by the publisher.

## References

[1] Kanoff J Sobrin L. New diagnosis and treatment paradigms in acute retinal necrosis. Int Ophthalmol Clin. (2011) 51:25–31. 10.1097/IIO.0b013e31822d6864 21897137

[2] Cochrane TF Silvestri G McDowell C Foot B McAvoy CE. Acute retinal necrosis in the United Kingdom: results of a prospective surveillance study. Eye. (2012) 26:370–7. 10.1038/eye.2011.338 22281865 PMC3298997

[3] Standardization of Uveitis Nomenclature (SUN) Working Group. Classification criteria for acute retinal necrosis syndrome. Am J Ophthalmol. (2021) 228:237–44. 10.1016/j.ajo.2021.03.057 33845012 PMC8675365

[4] Kolacny D Stalmans P Wouters C Van Ranst M Casteels I. Bilateral acute retinal necrosis in a 12-year-old girl. J AAPOS. (2005) 9:599–601. 10.1016/j.jaapos.2005.07.010 16414533

[5] Luck S Sharland M Griffiths P Jenkins SM. Advances in the antiviral therapy of herpes virus infection in children. Expert Rev Anti Infect Ther. (2006) 4:1005–20. 10.1586/14787210.4.6.1005 17181417

[6] Silva RA Berrocal AM Moshfeghi DM Blumenkranz MS Sanislo S Davis JL. Herpes simplex virus type 2 mediated acute retinal necrosis in a pediatric population: case series and review. Graefes Arch Clin Exp Ophthalmol. (2013) 251:559–66. 10.1007/s00417-012-2164-8 23052715

[7] Chan EW Sun V Eldeeb M Kapusta MA. Epstein-Barr virus acute retinal necrosis in an immunocompetent host. Retin Cases Brief Rep. (2021) 15:412–6. 10.1097/ICB.0000000000000819 30358736

[8] Gallego-Pinazo R Harto M Garcia-Medina JJ Serra I España E Pinazo-Duran MD. Epstein-Barr virus and acute retinal necrosis in a 5-year-old immunocompetent child. Clin Ophthalmol. (2008) 2:451–5. 10.2147/OPTH.S1757 19668736 PMC2693969

[9] Pikkel YY Pikkel J. Acute retinal necrosis in childhood. Case Rep Ophthalmol. (2014) 5:138–43. 10.1159/000363130 24932179 PMC4049010

[10] Charles G. Acute retinal necrosis caused by herpes simplex virus type 2 in children: reactivation of an undiagnosed latent neonatal herpes infection. Semin Pediatr Neurol. (2012) 19:115–8. 10.1016/j.spen.2012.02.005 22889540 PMC3419358

[11] Ganatra JB Chandler D Santos C Kuppermann B Margolis TP. Viral causes of the acute retinal necrosis syndrome. Am J Ophthalmol. (2000) 129:166–72. 10.1016/S0002-9394(99)00316-5 10682968

[12] Van Gelder RN Willig JL Holland GN Kaplan HJ. Herpes simplex virus type 2 as a cause of acute retinal necrosis syndrome in young patients. Ophthalmology. (2001) 108:869–76. 10.1016/S0161-6420(01)00556-5 11320015

[13] Morse LS Mizoguchi M. Diagnosis and management of viral retinitis in the acute retinal necrosis syndrome. Semin Ophthalmol. (2009) 10:28–41. 10.3109/08820539509059977 10155697

[14] Tan JCH Byles D Stanford MR Frith PA Graham EM. Acute retinal necrosis in children caused by herpes simplex virus. Retina. (2001) 21:344–7. 10.1097/00006982-200108000-00008 11508880

[15] Tran THC Rozenberg F Fillet AM Bodaghi B. Diagnostic and therapeutic management of a severe acyclovir-resistant acute retinal necrosis in a young child. Graefes Arch Clin Exp Ophthalmol. (2005) 243:266–8. 10.1007/s00417-004-0985-9 15378385

[16] Landry ML Mullangi P Nee P Klein BR. Herpes simplex virus type 2 acute retinal necrosis 9 years after neonatal herpes. J Pediatr. (2005) 146:836–8. 10.1016/j.jpeds.2005.02.025 15973328

[17] Khurana RN Charonis A Samuel MA Gupta A Tawansy KA. Intravenous foscarnet in the management of acyclovir-resistant herpes simplex virus type 2 in acute retinal necrosis in children. Medical Sci Monit. (2005) 11:CS75–8. 16319793

[18] King J Chung M DiLoreto DA. A 9 year-old girl with herpes simplex virus type 2 acute retinal necrosis treated with intravitreal foscarnet. Ocul Immunol Inflamm. (2007) 15:395–8. 10.1080/09273940701486431 17972224

[19] Chiquet C Bodaghi B Mougin C Najioullah F. Acute retinal necrosis diagnosed in a child with chronic panuveitis. Graefes Arch Clin Exp Ophthalmol. (2006) 244:1206–8. 10.1007/s00417-005-0233-y 16411098

[20] Tanaka-Kitajima N Iwata N Ando Y Sakurai H Iwami M Tsuzuki K. Acute retinal necrosis caused by herpes simplex virus type 2 in a 3-year-old Japanese boy. Eur J Pediatr. (2009) 168:1125–8. 10.1007/s00431-008-0878-8 19050917

[21] Gupta A Rani PK Bagga B Dore P Mittal A Jalali S. Bilateral herpes simplex-2 acute retinal necrosis with encephalitis in premature twins. J AAPOS. (2010) 14:541–3. 10.1016/j.jaapos.2010.08.011 21168079

[22] Hsu C Moinfar N Lipman B Capone A Trese M. Acute retinal necrosis in a neonate. Retin Cases Brief Rep. (2013) 7:406–8. 10.1097/ICB.0b013e318297f6d5 25383815

[23] Ren ZX Xu F Yao ZW Li BY Chang YQ Zhang ZY. Acute retinal necrosis in a neonate with HSV II encephalitis. Pediatr Neonatol. (2019) 60:344–5. 10.1016/j.pedneo.2018.06.001 30122364

[24] Tran THC Stanescu D Caspers-Velu L Rozenberg F Liesnard C Gaudric A. Clinical characteristics of acute HSV-2 retinal necrosis. Am J Ophthalmol. (2004) 137:872–9. 10.1016/j.ajo.2003.12.036 15126152

[25] Chou HD Teh WM Sun MH Chen KJ. Epstein-Barr virus retinitis in an immunocompromised child: a case report. Eur J Ophthalmol. (2020). 10.1177/1120672120926855 32460537

[26] Choi SI Kim JR Ra H. Necrotizing herpetic retinopathy in an immune-compromised pediatric patient with minimal signs of inflammation: case report. BMC Ophthalmol. (2016) 16:85. 10.1186/s12886-016-0253-x 27277425 PMC4898446

[27] Smith LK Kurz PA Wilson DJ Flaxel CJ Rosenbaum JT. Two patients with the von Szily reaction: herpetic keratitis and contralateral retinal necrosis. Am J Ophthalmol. (2007) 143:536–8. 10.1016/j.ajo.2006.10.040 17317412

[28] Kim SJ Kang SW Joo EY. An unusual case of herpes simplex viral encephalitis following acute retinal necrosis after administration of a systemic steroid. J Epilepsy Res. (2012) 2:21–4. 10.14581/jer.12006 24649457 PMC3952316

[29] Vandercam T Hintzen RQ de Boer JH Van der Lelij A. Herpetic encephalitis is a risk factor for acute retinal necrosis. Neurology. (2008) 71:1268–74. 10.1212/01.wnl.0000327615.99124.99 18852442

[30] Okunuki Y Usui Y Kezuka T Takeuchi M Goto H. Four cases of bilateral acute retinal necrosis with a long interval after the initial onset. Br J Ophthalmol. (2011) 95:1251–4. 10.1136/bjo.2010.191288 21242577

[31] Atherton SS Cathcart HM. Anterior segment mechanisms of protection during herpes simplex virus 1 infection. Jpn J Ophthalmol. (2010) 54:182–6. 10.1007/s10384-010-0798-9 20577848 PMC4021309

[32] Marshall JD Heeke DS Abbate C Yee P Van Nest G. Induction of interferon-gamma from natural killer cells by immunostimulatory CpG DNA is mediated through plasmacytoid-dendritic-cell-produced interferon-alpha and tumour necrosis factor-alpha. Immunology. (2006) 117:38–46. 10.1111/j.1365-2567.2005.02261.x 16423039 PMC1782206

[33] Zheng M Atherton SS. Cytokine profiles and inflammatory cells during HSV-1-induced acute retinal necrosis. Invest Ophthalmol Vis Sci. (2005) 46:1356–63. 10.1167/iovs.04-1284 15790902

[34] Zhao XY Meng LH Zhang WF Wang DY Chen YX. Retinal detachment following acute retinal necrosis and the efficacies of different interventions: a systematic review and meta-analysis. Retina. (2020) 41:965–78. 10.1097/IAE.0000000000002971 32932382

[35] Lau CH Missotten T Salzmann J Lightman SL. Acute retinal necrosis features, management, and outcomes. Ophthalmology. (2007) 114:756–62. 10.1016/j.ophtha.2006.08.037 17184841

[36] Han DP Lewis H Williams GA Mieler WF Abrams GW Aaberg TM. Laser photocoagulation in the acute retinal necrosis syndrome. Arch Ophthalmol. (1987) 105:1051–4. 10.1001/archopht.1987.01060080053027 3632413

